# Supplementary material for: Laser-pulse-shape control of seeded QED cascades
Source: Sci Rep. 2017 Jul 18;7:5694. doi: 10.1038/s41598-017-05891-z (PMC5516046; doi:10.1038/s41598-017-05891-z)
Supplement: Supplementary file 1 — Supplementary Information [file 41598_2017_5891_MOESM1_ESM.pdf]

Laser-pulse-shape control of seeded QED cascades: Supplementary information

Matteo Tamburini[1,*], Antonino Di Piazza[1], and Christoph H. Keitel[1]

[1]Max-Planck-Institut für Kernphysik, Saupfercheckweg 1, D-69117 Heidelberg, Germany
[*]matteo.tamburini@mpi-hd.mpg.de

Our implementation of stochastic photon emission by an electron or a positron follows a similar approach as Ref.[1], except that no lower energy cut-off is introduced for the emission of soft photons. First, at each time step Δt the electron/positron quantum parameter $\chi_{e/p}$ is calculated according to:

$$\chi_{e/p} = \frac{e\hbar}{m_e^3 c^4}\sqrt{\left(\frac{\varepsilon_{e/p}\mathbf{E}}{c} + \mathbf{p}_{e/p}\times\mathbf{B}\right)^2 - \left(\mathbf{p}_{e/p}\cdot\mathbf{E}\right)^2} \tag{1}$$

Then, the total probability of emitting a photon:

$$W_{\mathrm{rad},e/p} = \int_0^{\varepsilon_{e/p}} \frac{dW_{\mathrm{rad},e/p}(\varepsilon_\gamma)}{d\varepsilon_\gamma}\, d\varepsilon_\gamma \tag{2}$$

is calculated, where $dW_{\mathrm{rad},e/p}(\varepsilon_\gamma)/d\varepsilon_\gamma$ is the differential probability for an electron/positron with energy $\varepsilon_{e/p}$ to emit a photon with energy ε_γ is (see Eq. (4.24) in Ref.[2]):

$$\frac{dW_{\mathrm{rad},e/p}(\varepsilon_\gamma)}{d\varepsilon_\gamma} = \frac{\alpha m_e^2 c^4}{\sqrt{3}\pi\hbar\varepsilon_{e/p}^2}\frac{1}{(1+u_{e/p})}\left\{\left[1+(1+u_{e/p})^2\right]\mathrm{K}_{2/3}\left(\frac{2u_{e/p}}{3\chi_{e/p}}\right) - (1+u_{e/p})\int_{2u_{e/p}/3\chi_{e/p}}^{\infty}\mathrm{K}_{1/3}(y)dy\right\} \tag{3}$$

where $u_{e/p} = \varepsilon_\gamma/(\varepsilon_{e/p}-\varepsilon_\gamma)$ and $\mathrm{K}_\nu(x)$ are the modified Bessel functions of the second kind. For each electron and positron a photon emission occurs if $r_1 < W_{\mathrm{rad},e/p}\Delta t$, where $0 < r_1 < 1$ is a uniformly distributed random number being generated at each time step. If the above-mentioned condition is fulfilled, the energy of the emitted photon ε_γ is obtained as the root of the sampling equation:

$$\int_0^{\varepsilon_\gamma} \frac{dW_{\mathrm{rad},e/p}(\varepsilon_\gamma)}{d\varepsilon_\gamma}\, d\varepsilon_\gamma = r_2 W_{\mathrm{rad},e/p} \tag{4}$$

where $0 < r_2 < 1$ is a uniformly distributed random number independent of r_1 being generated at each photon creation event. The direction of propagation of the emitted photon is parallel to the momentum $\mathbf{p}_{e/p}$ of the parental electron/positron, and the time step is chosen such that the condition $W_{\mathrm{rad},e/p}\Delta t \ll 1$ holds. Note that $dW_{\mathrm{rad},e/p}(\varepsilon_\gamma)/d\varepsilon_\gamma$ diverges as $dW_{\mathrm{rad},e/p}(\varepsilon_\gamma)/d\varepsilon_\gamma = \mathscr{O}\left(\varepsilon_\gamma^{-2/3}\right)$ for $\varepsilon \to 0$, but its integral $\int_0^{\varepsilon_\gamma} d\varepsilon_\gamma dW_{\mathrm{rad},e/p}(\varepsilon_\gamma)/d\varepsilon_\gamma$ is convergent. By changing the integration variable from ε_γ to w with the substitution $\varepsilon_\gamma = w^3$ one obtains a well-behaved continuous function which can be integrated numerically in the whole interval $0 < \varepsilon_\gamma < \varepsilon_{e/p}$.

The event generator for the creation of an electron-positron pair from a hard-photon follows the same steps as for the photon emission, except that $dW_{\mathrm{rad},e/p}(\varepsilon_\gamma)/d\varepsilon_\gamma$ is replaced by the differential probability for a photon with energy ε_γ to convert into an electron-positron pair $dW_{\mathrm{pair}}(\varepsilon_e)/d\varepsilon_e$, which is given by (see Eq. (3.50) in Ref.[2]):

$$\frac{dW_{\mathrm{pair}}(\varepsilon_e)}{d\varepsilon_e} = \frac{\alpha m_e^2 c^4}{\sqrt{3}\pi\hbar\varepsilon_\gamma^2}\left[\frac{\varepsilon_e^2+\varepsilon_p^2}{\varepsilon_e\varepsilon_p}\mathrm{K}_{2/3}\left(\frac{2\varepsilon_\gamma^2}{3\chi_\gamma\varepsilon_e\varepsilon_p}\right) + \int_{2\varepsilon_\gamma^2/3\chi_\gamma\varepsilon_e\varepsilon_p}^{\infty} dy\mathrm{K}_{1/3}(y)\right] \tag{5}$$

where ε_e is the electron energy ($0 < \varepsilon_e < \varepsilon_\gamma$) and $\varepsilon_p = (\varepsilon_\gamma - \varepsilon_e)$ is the positron energy.

Figure 1 displays the photon spectrum $dI/d\varepsilon_\gamma = \varepsilon_\gamma dW_{\mathrm{rad},e/p}(\varepsilon_\gamma)/d\varepsilon_\gamma$ (red dotted line) obtained from 2×10^7 photon emissions by electrons with $\varepsilon_e = 100\,m_ec^2$ in a constant and uniform magnetic field $B = 10^{-2}F_{\mathrm{cr}}$ such that $\chi_e = 1$, and the corresponding analytical result from Eq. (3) (solid black line). Figure 2 displays the electron energy distribution $dW_{\mathrm{pair}}(\varepsilon_e)/d\varepsilon_e$ (red dotted line) obtained from 2×10^7 photon conversion into electron-positron pairs by photons with $\varepsilon_\gamma = 100\,m_ec^2$ in

a constant and uniform magnetic field $B = 10^{-2} F_{\rm cr}$ such that $\chi_\gamma = 1$, and the corresponding analytical result from Eq. (5) (solid black line). In both cases excellent agreement between the numerical and the analytical results is obtained. In addition, as a benchmark for our code, the development of an avalanche-type cascade initiated by a single seed electron is simulated with the same parameters as in Fig. 12 of Ref.[3]. The seed electron collides with a standing wave generated by two counterpropagating plane waves such that at the initial instant $t = 0$ the electromagnetic field has only a single component $E_y(t = 0) = 10^{-2} F_{\rm cr} \sin(2\pi x/\lambda)$ and the electron has momentum $\mathbf{p}_e = (0, 10^3 m_e c, 0)$ and is located at $x = \lambda/8$, where $\lambda = 1\,\mu$m. Figure 3 displays the number of electrons as function of time obtained from 10^2 independent runs. The solid red line corresponds to the average result, the dotted black lines denote the region within one standard deviation from the average, while the dashed blue lines correspond the maximal and minimal values obtained in the simulations. Finally, the thick solid grey line corresponds to the result reported in Fig. 12 of Ref.[3]. A fair agreement between our results and those of Ref.[3] is obtained.

References

1. Elkina, N. V. *et al.* QED cascades induced by circularly polarized laser fields. *Phys. Rev. ST Accel. Beams* **14**, 054401 (2011).
2. Baier, V. N., Katkov, V. M. & Strakhovenko, V. M. *Electromagnetic Processes at High Energies in Oriented Single Crystals* (World Scientific, Singapore, 1998).
3. Gonoskov, A. *et al.* Extended particle-in-cell schemes for physics in ultrastrong laser fields: Review and developments. *Phys. Rev. E* **92**, 023305 (2015).

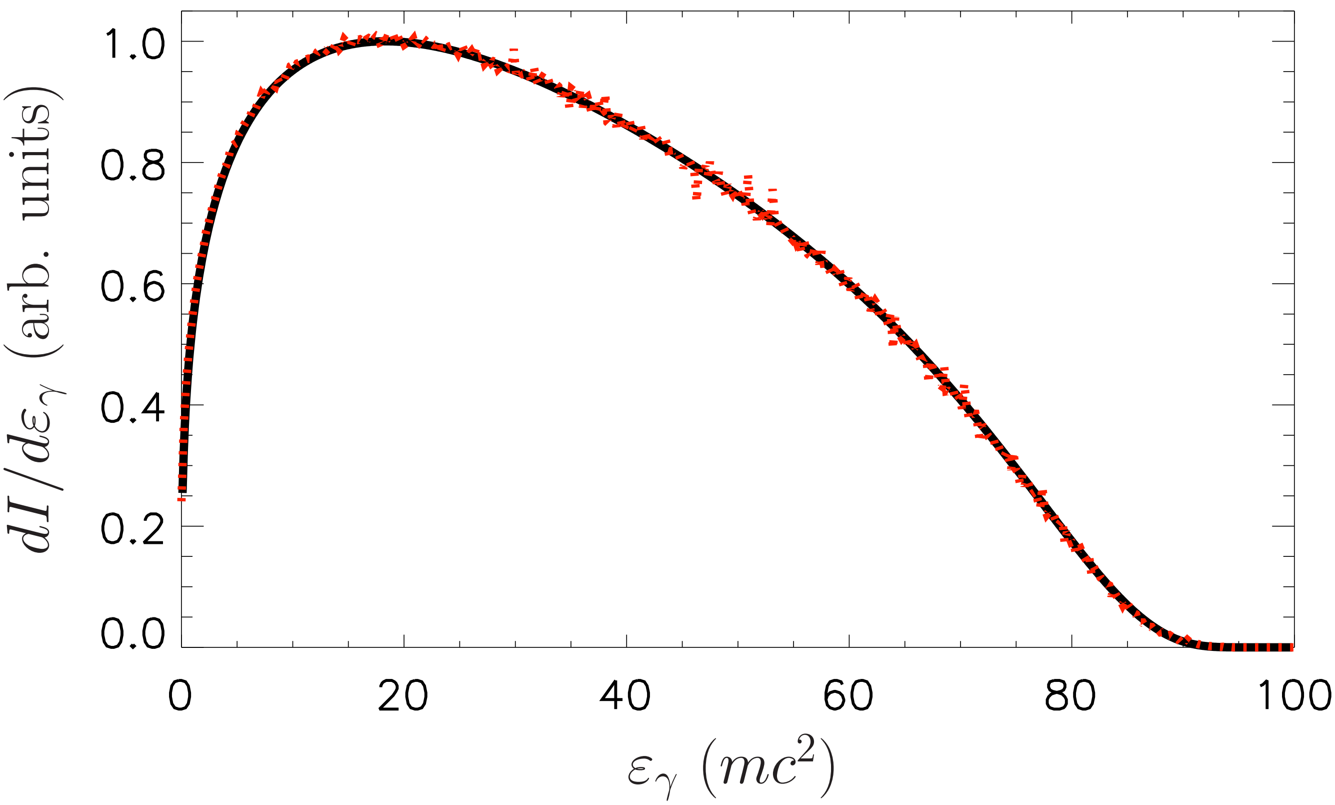

Figure 1. The photon spectrum $dI/d\varepsilon_\gamma = \varepsilon_\gamma dW_{{\rm rad},e/p}/d\varepsilon_\gamma$ (red dotted line) obtained from 2×10^7 photon emissions by electrons with $\varepsilon_e = 100 m_e c^2$ in a constant and uniform magnetic field $B = 10^{-2} F_{\rm cr}$ such that $\chi_e = 1$, and the corresponding analytical result (solid black line).

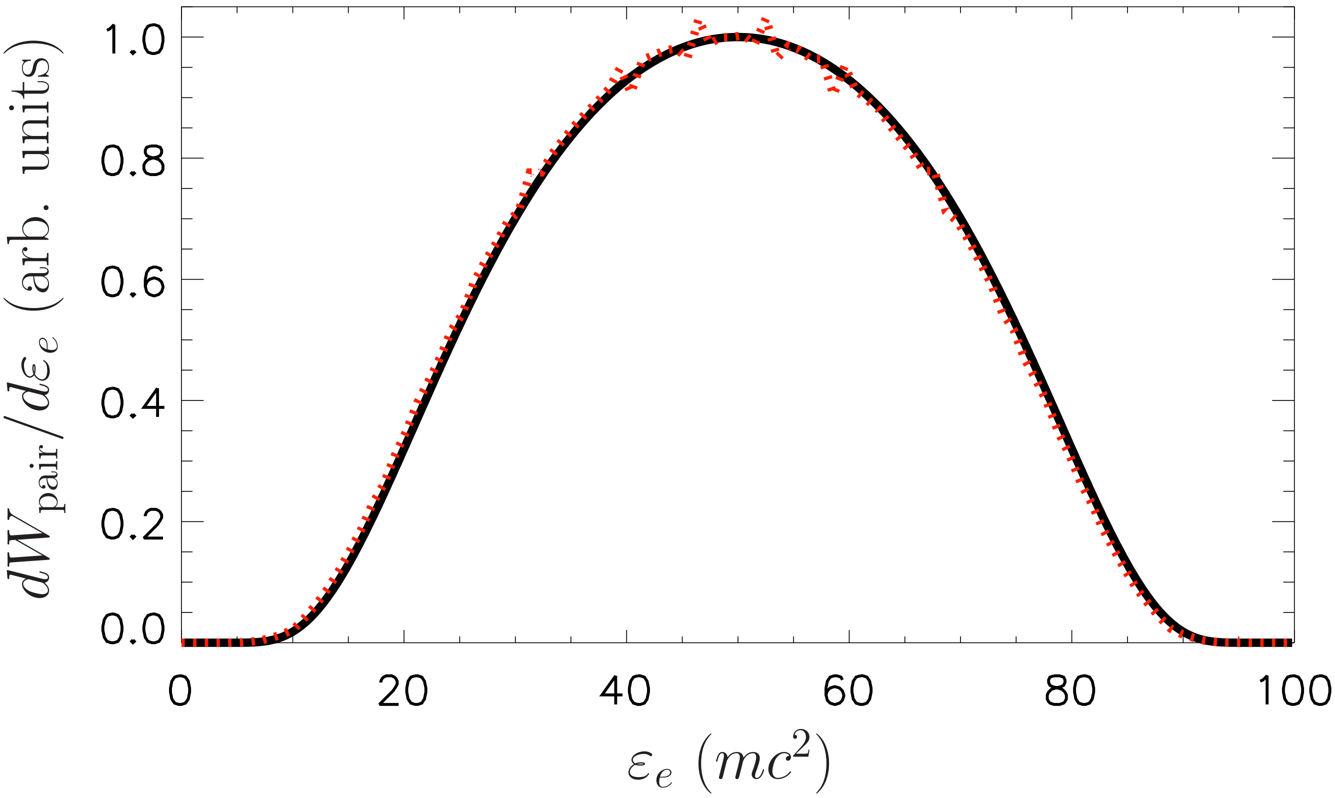

Figure 2. The electron energy distribution $dW_{\mathrm{pair}}/d\varepsilon_e$ (red dotted line) obtained from 2×10^7 photon conversion into electron-positron pairs by photons with $\varepsilon_\gamma = 100\, m_e c^2$ in a constant and uniform magnetic field $B = 10^{-2} F_{\mathrm{cr}}$ such that $\chi_\gamma = 1$, and the corresponding analytical result (solid black line).

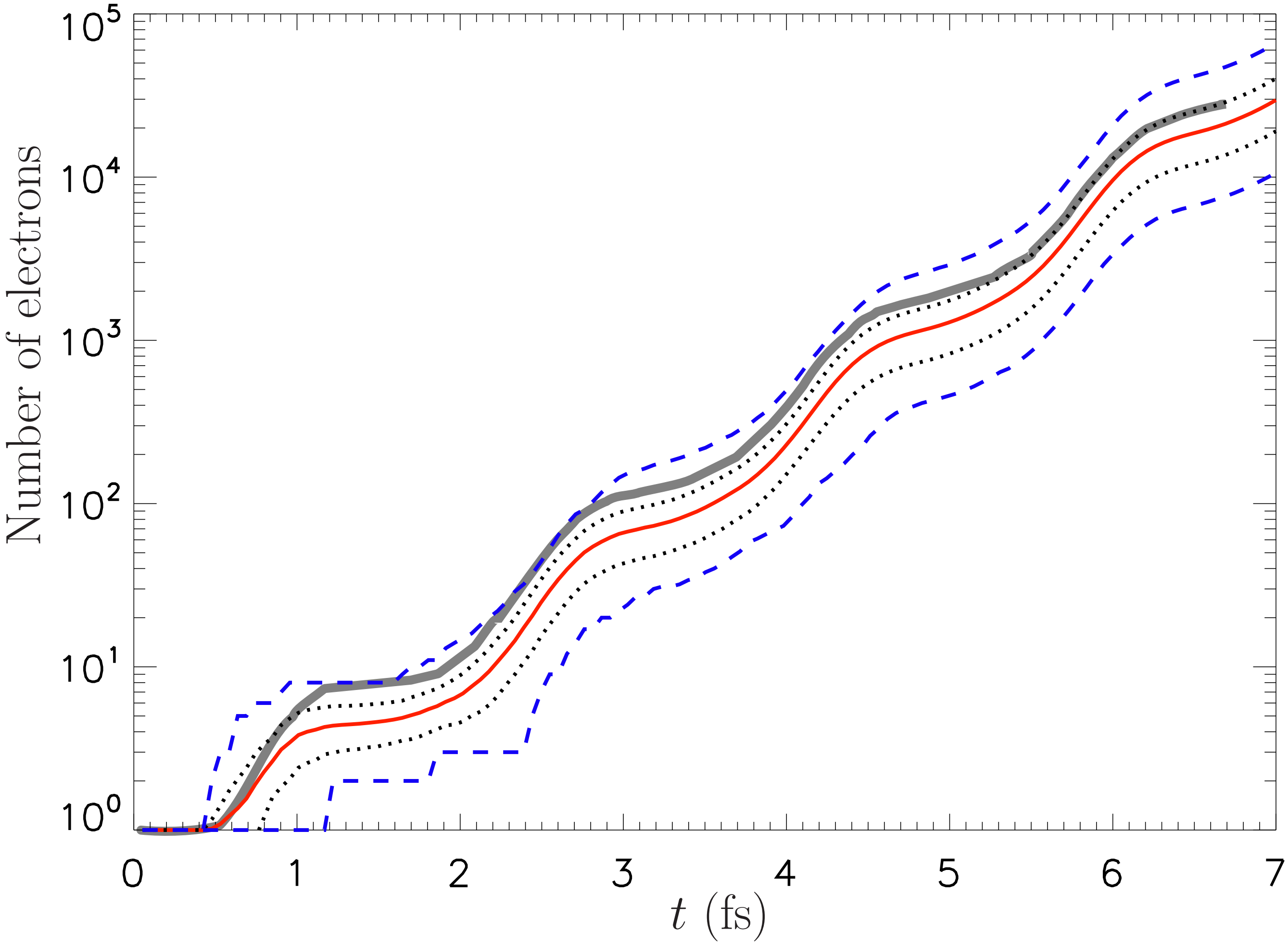

Figure 3. The number of electrons as function of time in a cascade initiated by a single seed electron colliding with a standing wave generated by two counterpropagating plane waves. At the initial instant $t = 0$, the electromagnetic field has only a single component $E_y(t = 0) = 10^{-2} F_{\rm cr} \sin(2\pi x/\lambda)$ and the electron has momentum $\mathbf{p}_e = (0, 10^3\, m_e c, 0)$ and is located at $x = \lambda/8$, where $\lambda = 1\,\mu$m. The figure shows: (i) the result averaged over 10^2 independent runs (red solid line), (ii) the region within one standard deviation from the average (black dotted lines), (iii) the maximal and minimal values obtained (blue dashed lines), (iv) the corresponding result obtained by Ref.[3] (thick solid grey line).
